# Supplementary material for: Non-redundant roles for the human mRNA decapping cofactor paralogs DCP1a and DCP1b
Source: Life Sci Alliance. 2024 Sep 10;7(11):e202402938. doi: 10.26508/lsa.202402938 (PMC11387620; doi:10.26508/lsa.202402938)

Evaluation of DCP1a and DCP1b KO cell lines.
WB showing the expression of DCP1a (left side) and DCP1b (right side) in these cells: M17 (control), HCT (control), A3 (single cell clone DCP1a KO), A9 (polyclonal cells DCP1a KO), B2 (single cell clone DCP1b), and B18 (single cell clone DCP1b KO). Two different exposures are shown: the lower exposure on the left and the higher on the right.

Entire blot was probed for either DCP1a or DCP1b. Two different exposures are shown.

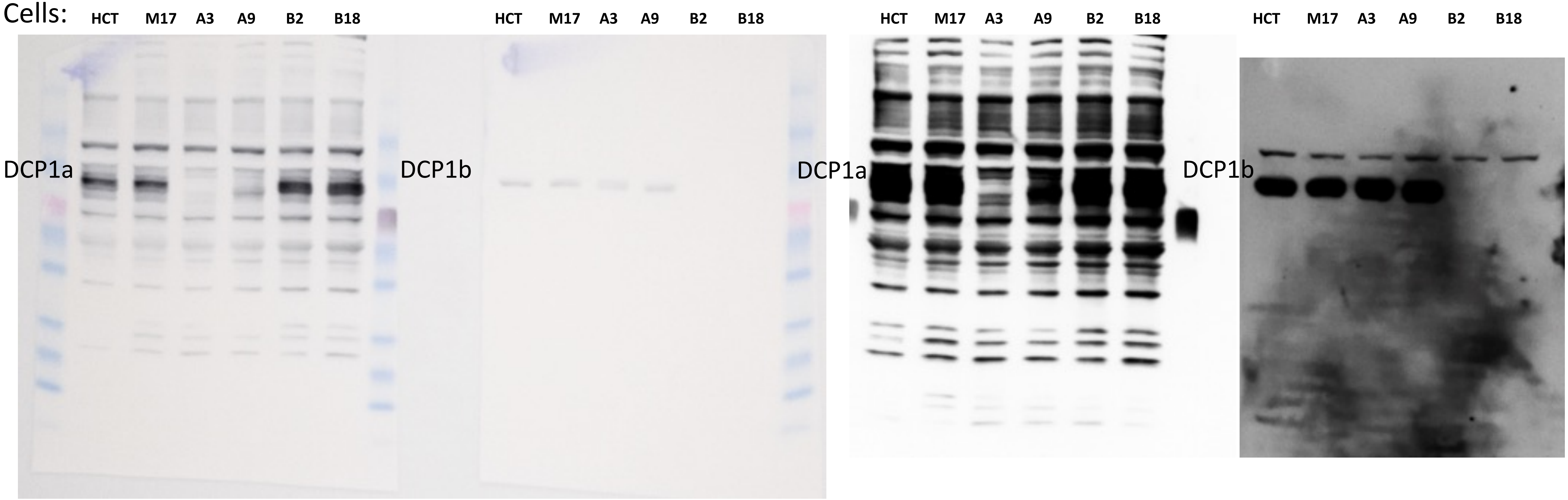

Evaluation of DCP1a and DCP1b depletion in clones A3, A9, B2, and B18. M17 and IgG are controls. A) Clones A3 and B2 were sent for profiling via LC/MS/MS. Intensity values are shown. Gel1, gel2, and gel3 are replicas 1, 2, and 3. There is no DCP1a or DCP1b expressed at the protein level in these clones. These samples were used for DDX6 IP in Figure 2. **B)** Clones A3 and B2 were sent for profiling via LS/MS/MS. Intensity values are shown; 1, 2, and 3 are replicas. DCP1a and DCP1b are not expressed at the protein level in these clones. These samples were used for the proteomics profiling in Figure 6. **C)** Intensities shown from clones B18 and A9 confirming B18 as a single cell clone. A9 is not a single cell clone, and there is still some DCP1a expressed in this cell line.

A

	Intensity A3-gel1	Intensity A3-gel2	Intensity A3-gel3	Intensity B2-gel1	Intensity B2-gel2	Intensity B2-gel3	Intensity IgG-gel1	Intensity IgG-gel2	Intensity IgG-gel3	Intensity M17-gel1	Intensity M17-gel2	Intensity M17-gel3
DCP1b	109390000	58371000	103530000	0	0	0	0	0	0	668270000	642910000	876890000
DCP1a	0	0	0	4451600000	4462800000	5544600000	14087000	4199400	16187000	4567600000	3973500000	4039300000

B

	Total Intensi	Intensity A3-1	Intensity A3-2	Intensity A3-3	Intensity B2-1	Intensity B2-2	Intensity B2-3	Intensity HCT-1	Intensity HCT-2	Intensity HCT-3
DCP1b	1.69E+07	2.81E+06	0	0	0	0	0	3.76E+06	3.17E+06	7.17E+06
DCP1a	2.44E+09	0	0	1.54E+07	4.27E+08	4.08E+08	4.11E+08	4.49E+08	3.53E+08	3.77E+08

C

	Intensity G1-M17IgG	Intensity G3-M17IgG	Intensity G1-B18-DCP1a	Intensity G3-B18-DCP1a	Intensity G1-M17-DCP1a	Intensity G3-M17-DCP1a	Intensity G2-M17-IgG	Intensity G4-M17-IgG	Intensity G2-A9-DCP1b	Intensity G4-A9-DCP1b	Intensity G2-M17-DCP1b	Intensity G4-M17-DCP1b
DCP1b	0	0	0	0	2025400000	1691500000	0	0	2824300000	1732300000	1761900000	2200000000
DCP1a	7754400	0	4205100000	7945300000	4658400000	4049500000	0	0	808500000	523270000	641440000	932590000

Evaluation of clones A9 and B18 with genomic PCR (gPCR):
A) Primers used are shown**.** #1 is forward primer, #2 is reverse primer. **B)** Agarose DNA gel of the gPCR with control DNA (M17), A9 clone (DCP1a KO), and B18 DNA (DCP1b KO). Abbreviations: A – DCP1a primer, B – DCP1b primer

A

DCP1a primers:

. Type or paste your sequence

Primer#1: 5'- CTCGATGGCACTTTCCTGTCCGC 25 nt: A=3.0 T=7.0 C=9.0 G=6.0 CG=60.00%

Primer#2: 5'- AATCCCAAGCGGACCCGACACC 22 nt: A=7.0 T=1.0 C=10.0 G=4.0 CG=63.64%

DCP1b primers:

. Type or paste your sequence

Primer#1: 5'- TCGCCGTGGGTTCTCGGTT 19 nt: A=0.0 T=7.0 C=5.0 G=7.0 CG=63.16%

Primer#2: 5'- ACTTCCAGGGCGTCAACGTCTCC 25 nt: A=4.0 T=6.0 C=10.0 G=5.0 CG=60.00%

B

M17 A9 M17 B18
primer A A B B

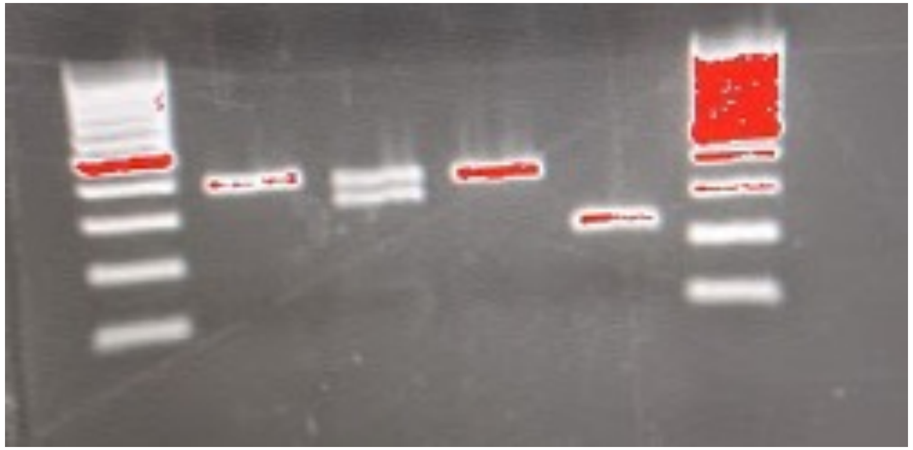

DNA Ladder DNA Ladder

M17 A3 M17 B2
primer A A B B

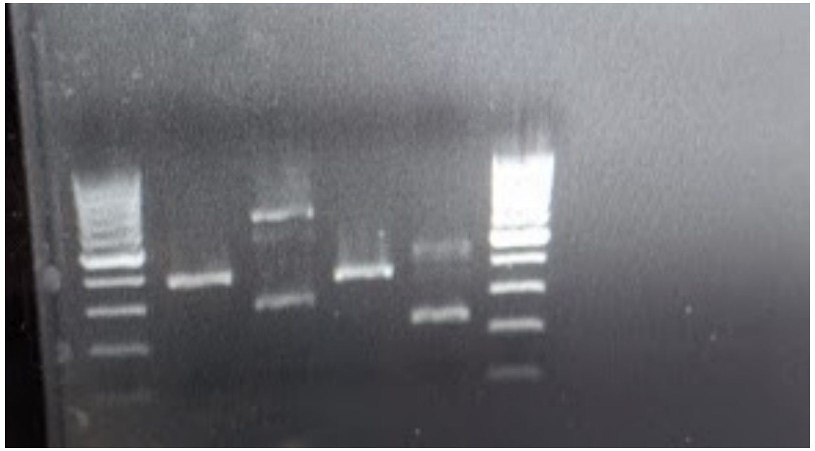

DNA Ladder DNA Ladder

Sequence alignment of the gPCR DNA of DCP1a KO (A9 clone) and DCP1a DNA sequence.

A9 clone FW primer

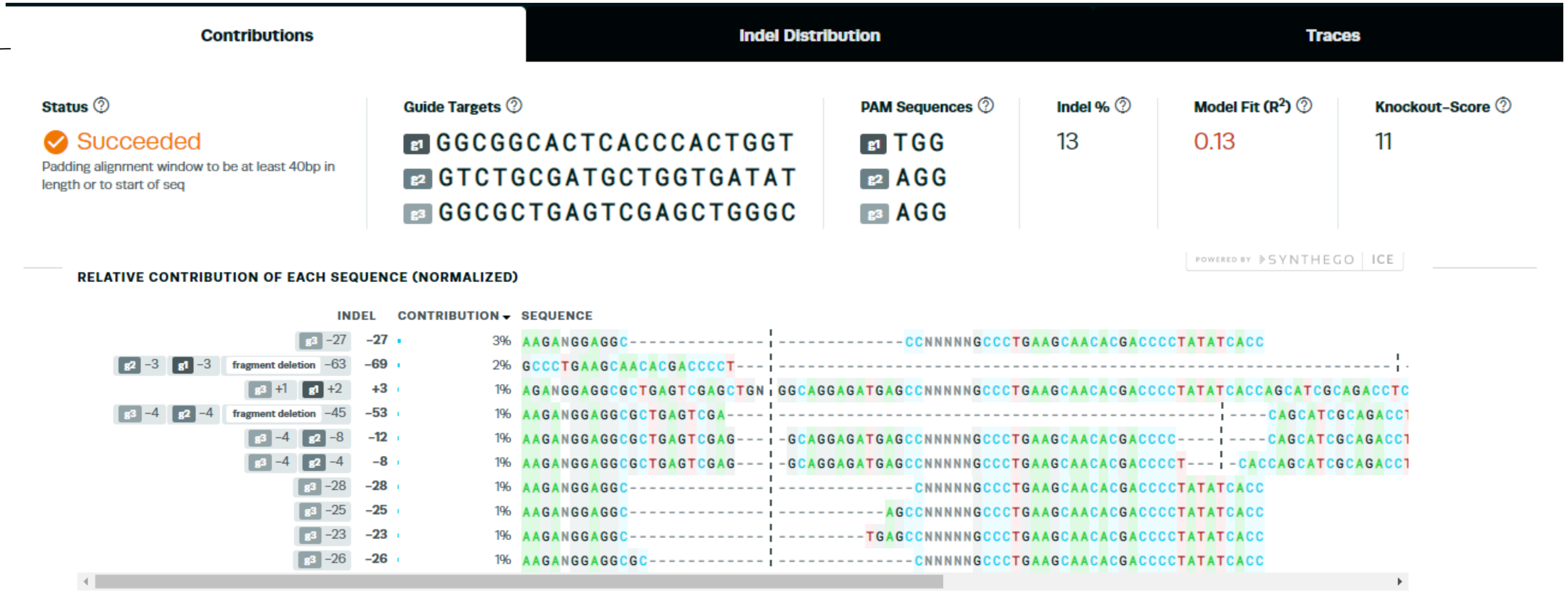

A9 clone RV primer

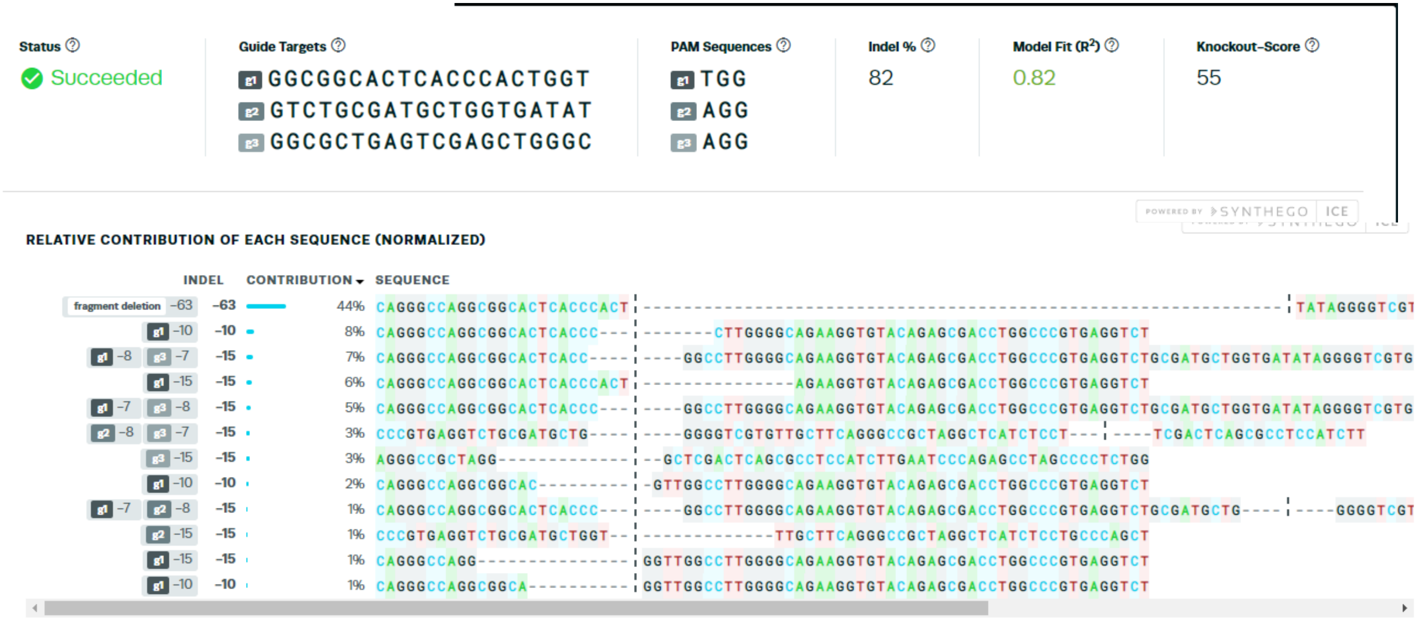

Sequence alignment of the gPCR DNA of DCP1a KO (A3 clone) and DCP1a DNA sequence.

Original DCP1a seq.

A3 gPCR sequence data with forward primer

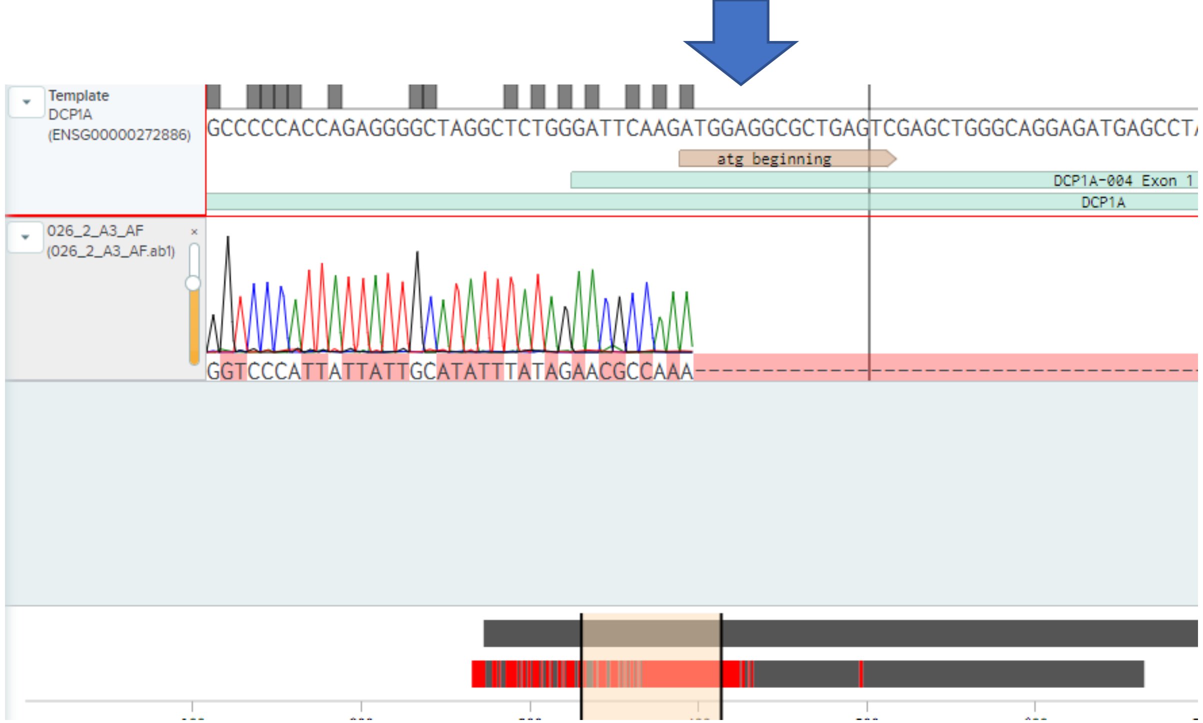

Original DCP1a seq.

A3 gPCR sequence data with reverse primer

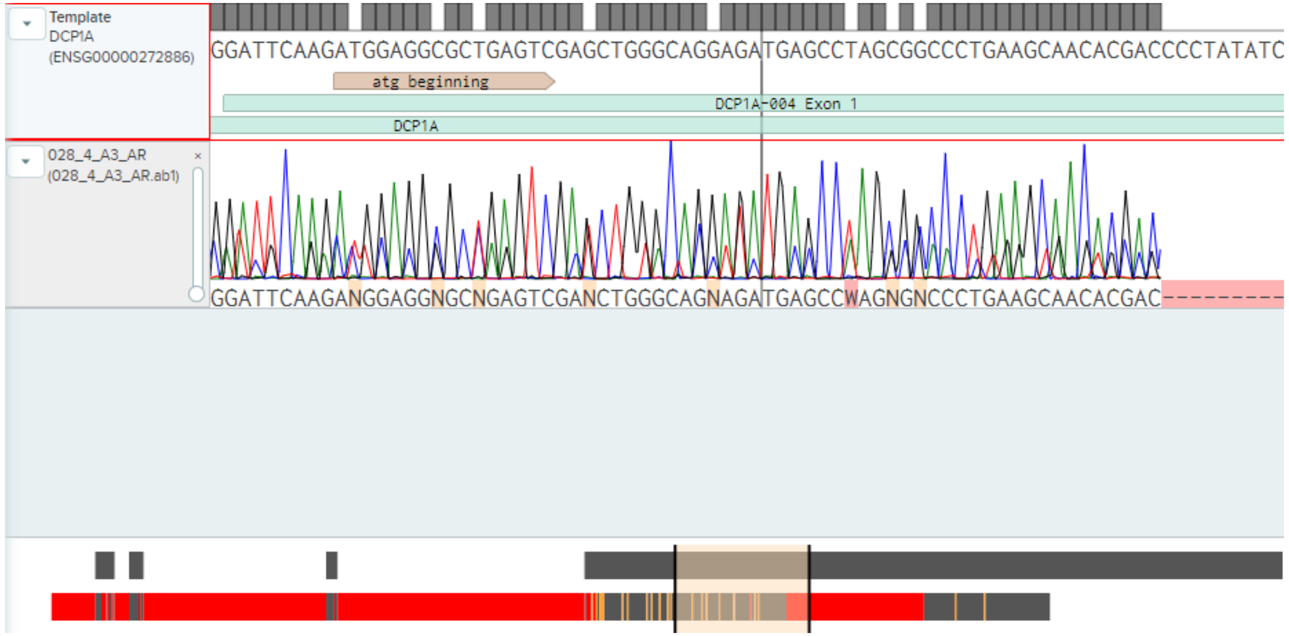

Sequence alignment of the and gPCR DNA of DCP1b KO (B18) and DCP1b DNA sequence.

DCP1b original sequence
B18 clone sequence

```
dcp1b      -NNNNNNNNNNNNNNTCCTGANTCTGANGCCCCCGCCTCCACCCCAACCCTGGGCTGCA 59
b18        NNNNNNNGGNNTCAGTCCTGANTCTGANNCCCCCGCCTCCACCCCAACCCTGGGCTGCA 60
            ******..**.  .*************.*******************************

dcp1b      CTGCTCCGCCGCGTNCGCACGCACCCACTCGTTGGCCCGATGGCCGAAGGTGTACAGAGC 119
b18        CTGCTCCGCCNCGTCCGCACGCACCCACT------------------------------- 89
           **********.*** **************

dcp1b      CACCTGGCTGGCCACGTCCACGATGCGGTTGATATAGGGGTCGTGGCGCTGCAGGGCCGC 179
b18        ------------------------------------------------------------ 89

dcp1b      TAGGCTGATGTCGCGCCCCTTTCCCACCAGGCCGCCTGCCGCCACGGCTGCCATCTTCCC 239
b18        --------------------------------------------CGGCTGCCATCTTCCC 105
                                                       ****************

dcp1b      TCCCTCCCAGACATAGGCACGGGGCTCTTGGAAGCCACTCTCAATAGATCGCAGAACGAG 299
b18        TCCCTCCCAGACATAGGCACGGGGCTCTTGGAAGCCACTCTCAATAGATCGCAGAACGAG 165
           ************************************************************

dcp1b      CGAGCTGCCTCGCAACCGAGAACCCACGGCNAN--------------------------- 332
b18        CGAGCTGCCTCGCAACCGAGAACCCACGGCGAANANNNNGNNNNNGNTTNCAGNAGCNTN 225
           ******************************.*

dcp1b      ------------------------------------------------------------ 332
b18        NNNNNNNGNNNNANNNNNGNNNNNNANNNNNNNGNNGGTGGNNNGNGGTNTGNNNNNNNN 285

dcp1b      ------------------------------------------------------------ 332
b18        AANNNNNNNNNCNGGGNNNNNNNNGNNNNGNNNNGCTNNNGNNNTANNGNACCNTGNNNN 345

dcp1b      -----------------------------------------------      332
b18        NNNNNNNNCCNNNNNNCTNNNNNANNNNNNGNNNNNNNNNNNNNNNN      392
```

Sequence alignment of the and gPCR DNA of DCP1b KO (B2 clone) and DCP1b DNA sequence.

Original dcp1b seq

B2 gPCR seq data with forward primer

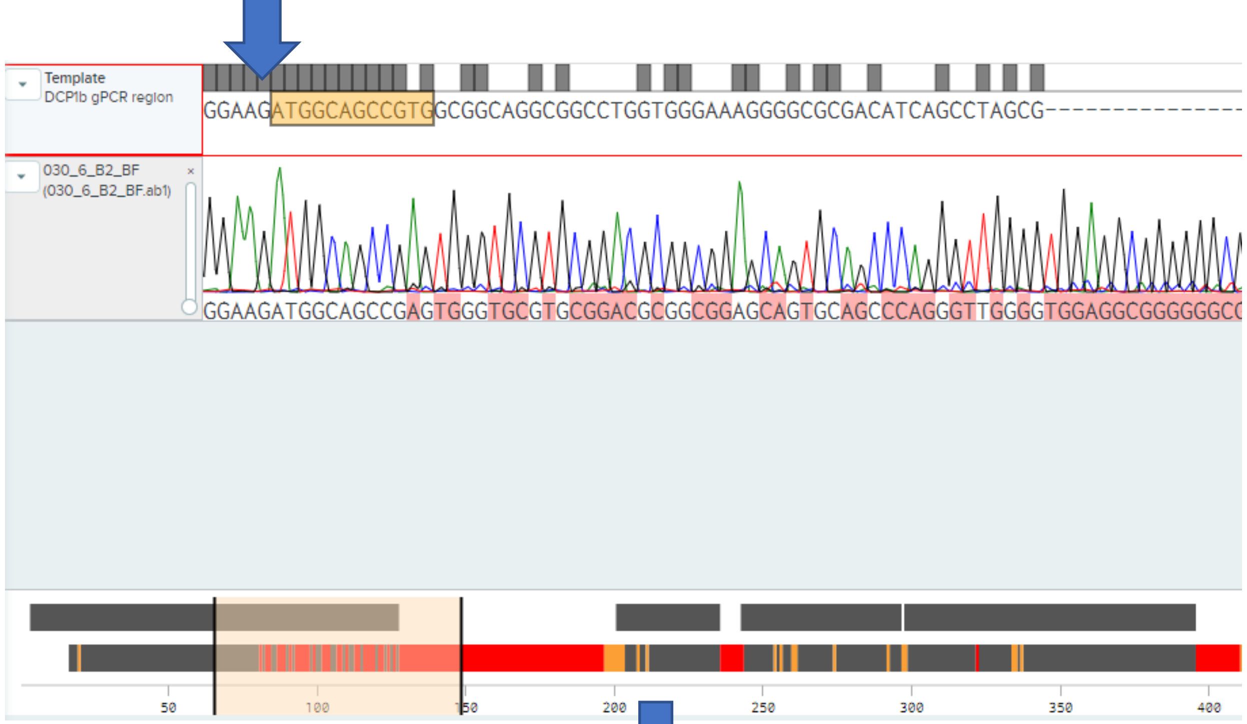

Original dcp1b seq

B2 gPCR seq data with reverse primer

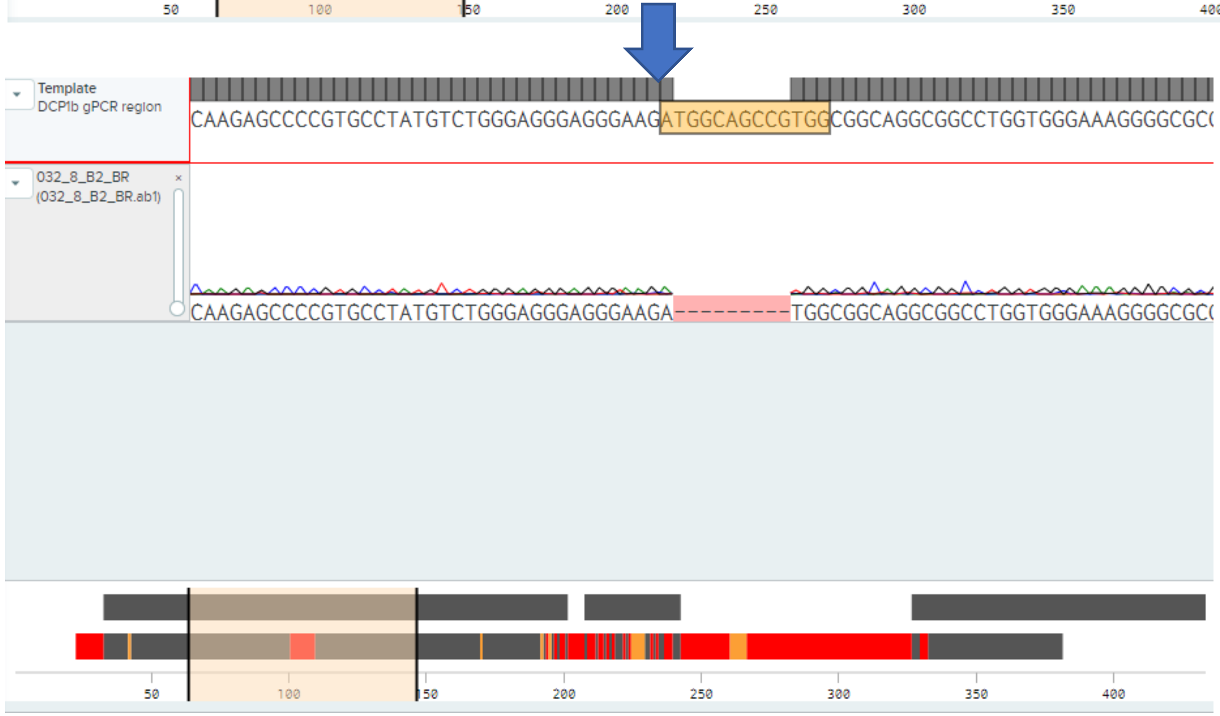

Sequence alignment of the p53 response element on DCP1b and the edited RE clones (RE1-3 and RE1-15). RE is indicated (top alignment with RE bar, bottom alignment highlighted section of the sequence)

RE1-15

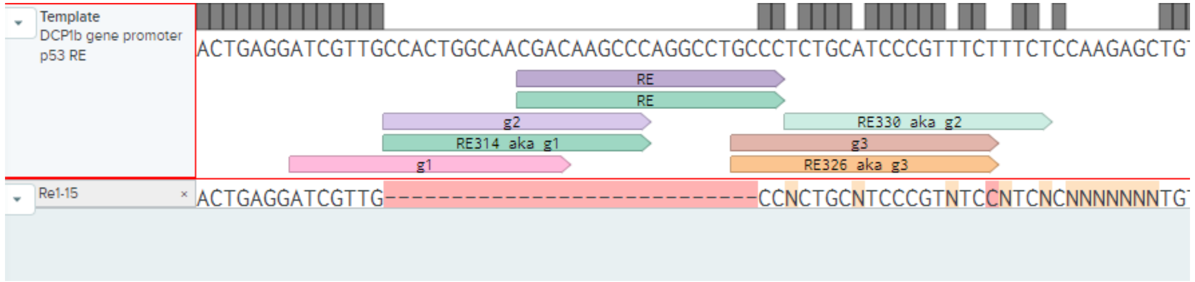

RE1-3

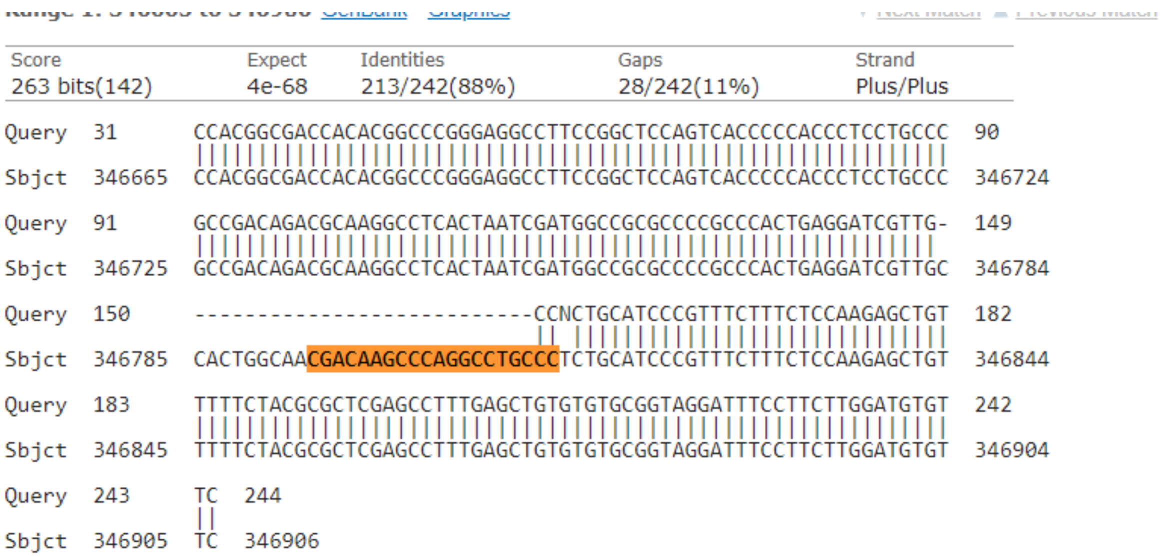

Supplement: Supplementary file 1 [file LSA-2024-02938_SdataFS2.pdf]
