# Supplementary material for: Non-redundant roles for the human mRNA decapping cofactor paralogs DCP1a and DCP1b
Source: Life Sci Alliance. 2024 Sep 10;7(11):e202402938. doi: 10.26508/lsa.202402938 (PMC11387620; doi:10.26508/lsa.202402938)

#replica 2 for figure 1B

HCT116 wt cells were infected with lentiviruses carrying shRNAs targeting LUC, DCP1b or DCP1a. On day two, the cells were selected with puromycin. On day five, cells were infected with the second virus expressing DCP1b or an empty vector control. Cells were harvested three days following the second round of infections and DCP2 was immunoprecipitated. Western blots (WB) showing inputs, IgG and DCP2 immunoprecipitation in shLUC, shDCP1a and shDCP1b cells.* Indicates that those protein bands where from a different SDS PAGE gel.

Abbreviations:
Untreated (Ut), pLenti vector (pL), overexpression of DCP1b (OE)
shLUC (Luc), shDCP1b (1b), shDCP1a (1a)

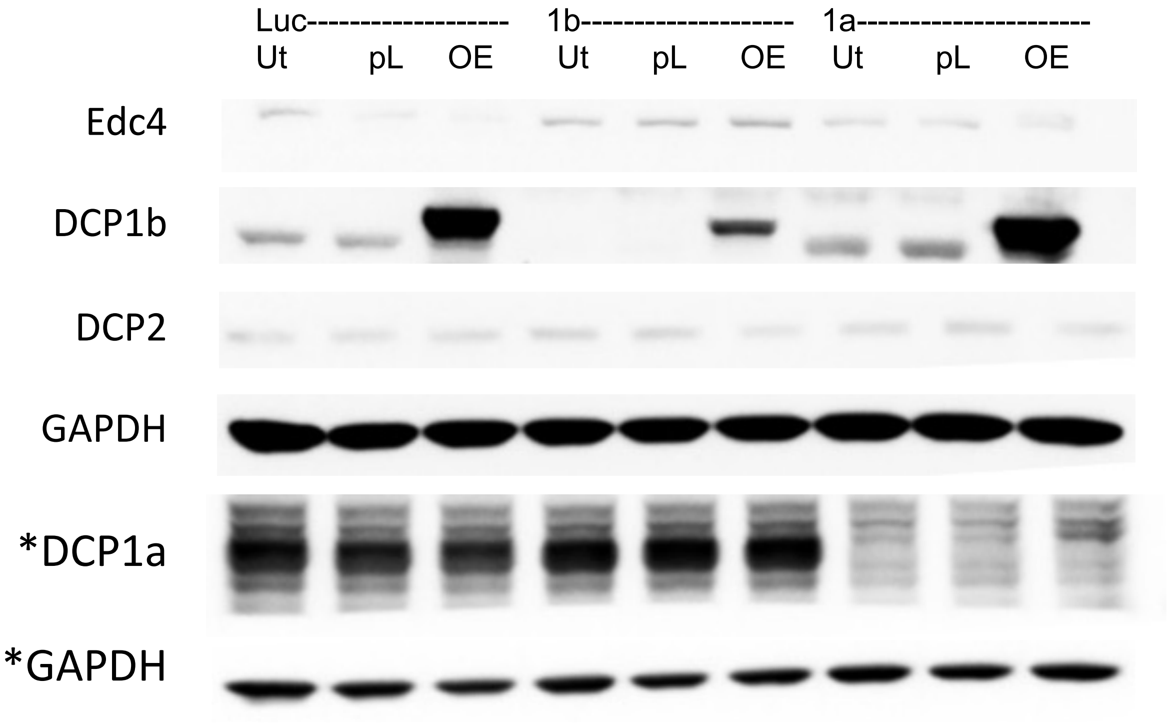

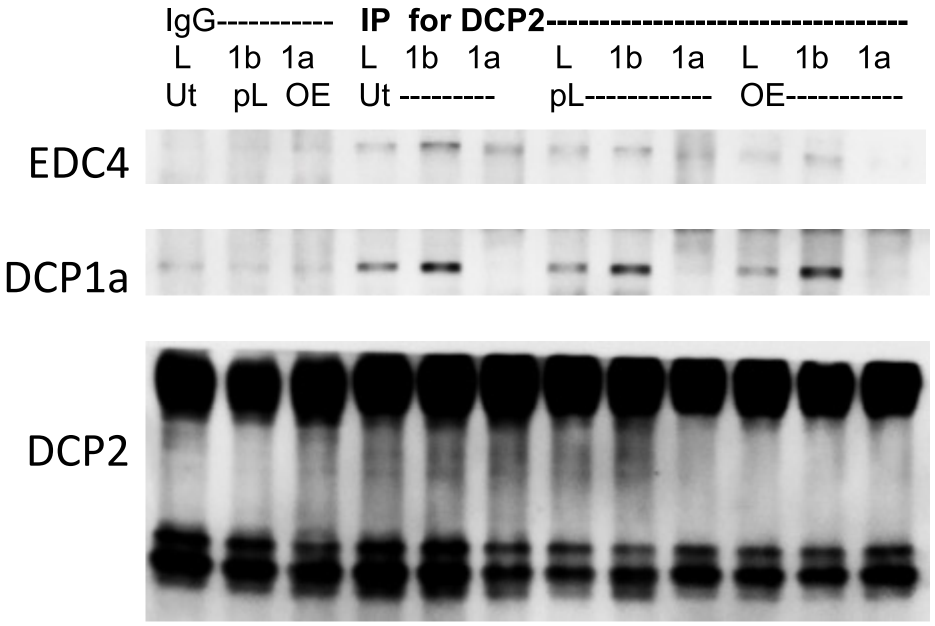

#replica 2 for Figure 1C:
HCT116 cells were transfected with Cas9 protein and no gRNA to create control cells or Cas9 protein and a mix of sgRNAs targeting either DCP1a or DCP1b. Three days post transfection single cell clones were selected and cultured for ten days. DCP2 was immunoprecipitated. WB showing inputs, IgG immunoprecipitation in control, DCP2 IP in Control (ctrl), DCP1a KO and DCP1b KO cells. * Indicates that those protein bands where from a different SDS PAGE gel.

Abbreviations:
M2, M4, M17 – crispr clones HCT116 wt cells were transfected with Cas9 protein and no guide RNA
A3, A15, A16 – crispr clones of DCP1a KO cells
B8, B18, B19 – crispr clones of DCP1b KO cells

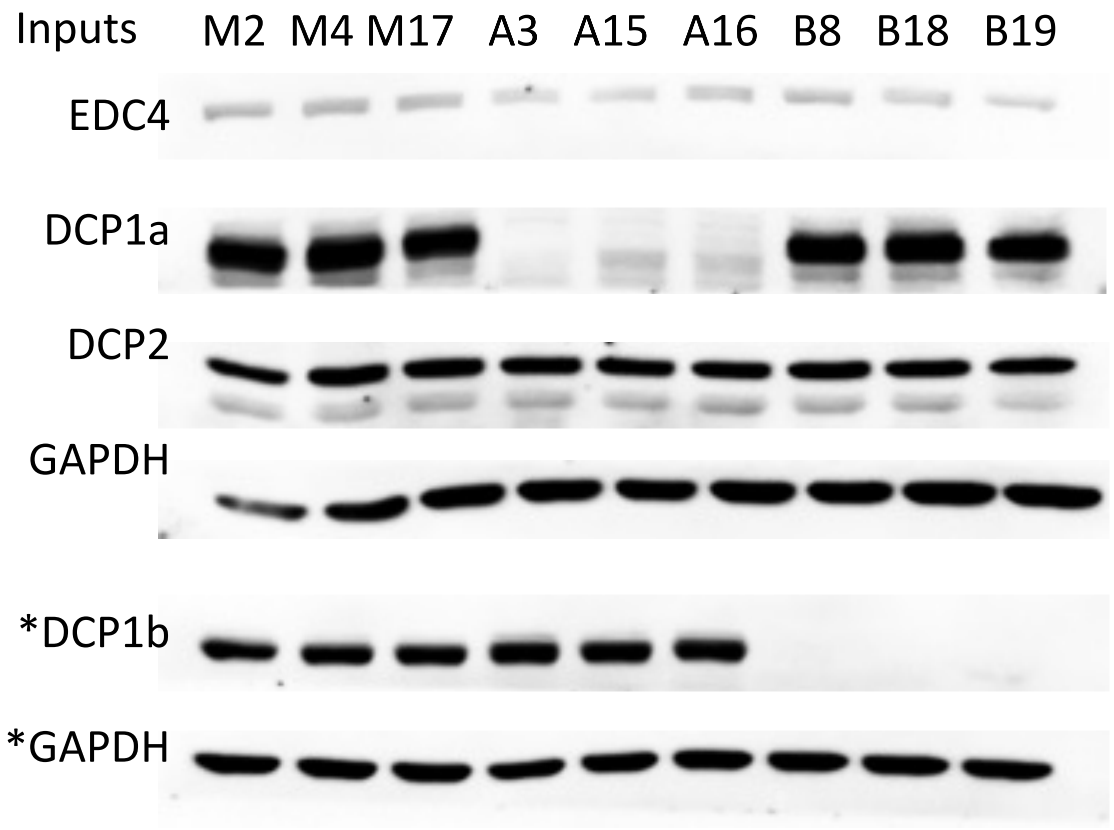

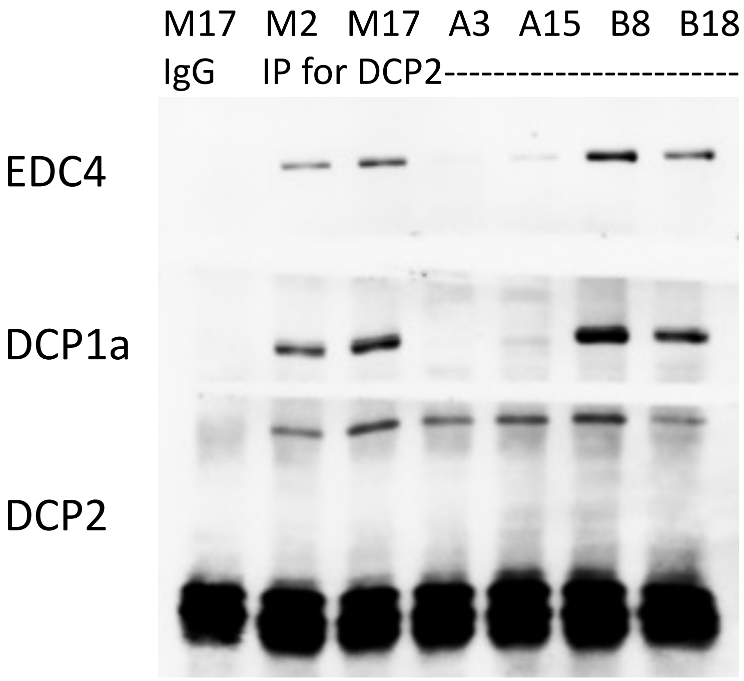

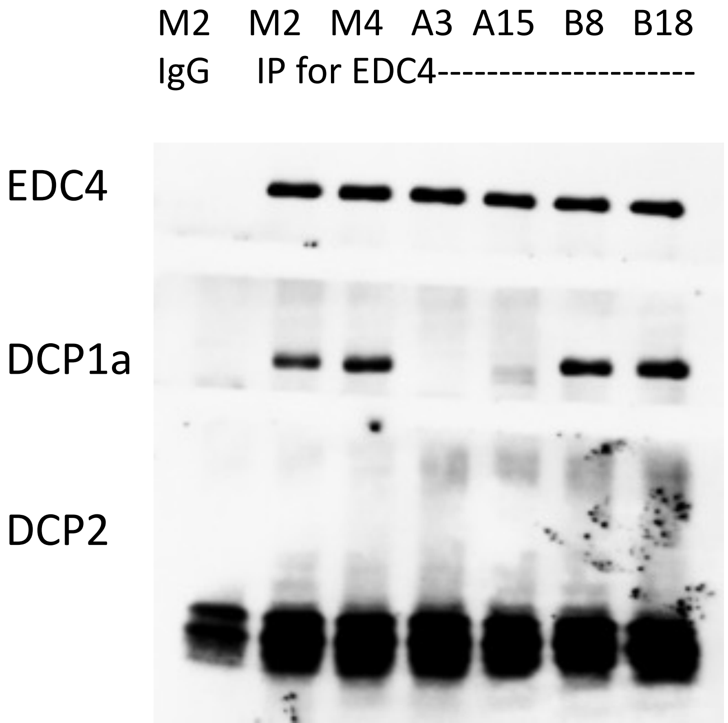

#replica 2 for Figure 1C:

#cont. replica for Figure 1C! Same samples as on the previous slide- same IP!

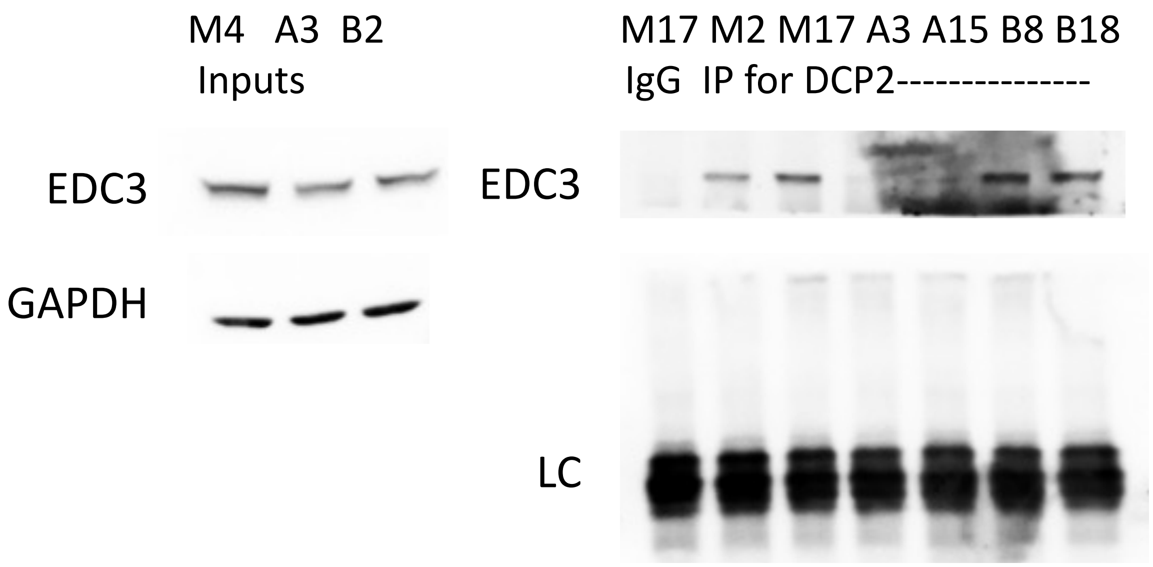

#replica 3 for Figure 1C:
HCT116 cells were transfected with Cas9 protein and no gRNA to create control cells or Cas9 protein and a mix of sgRNAs targeting either DCP1a or DCP1b. Three days post transfection single cell clones were selected and cultured for ten days. DCP2 was immunoprecipitated. WB showing inputs, IgG immunoprecipitation in control, DCP2 IP in Control (ctrl), DCP1a KO and DCP1b KO cells. * Indicates that those protein bands where from a different SDS PAGE gel.

Abbreviations:
M4, M17 – crispr clones HCT116 wt cells were transfected with Cas9 protein and no guide RNA
A3, A16 – crispr clones of DCP1a KO cells
B2, B18 – crispr clones of DCP1b KO cells

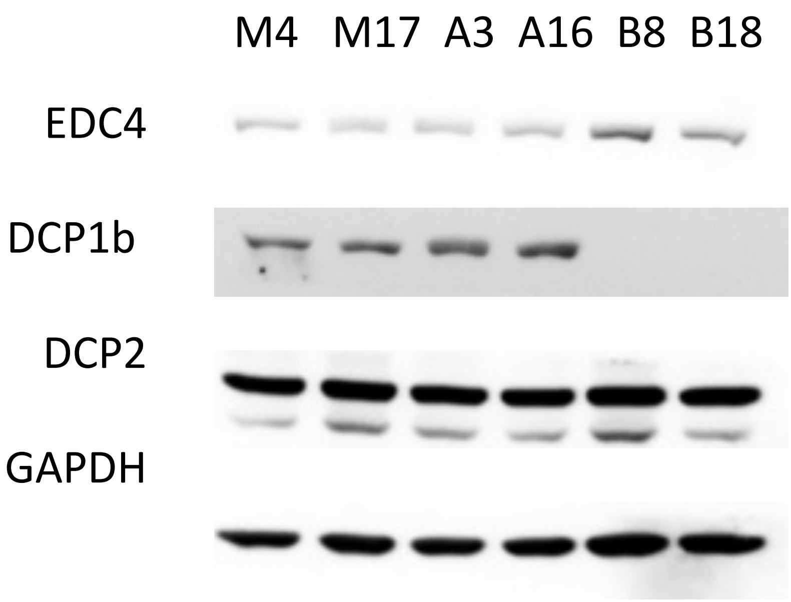

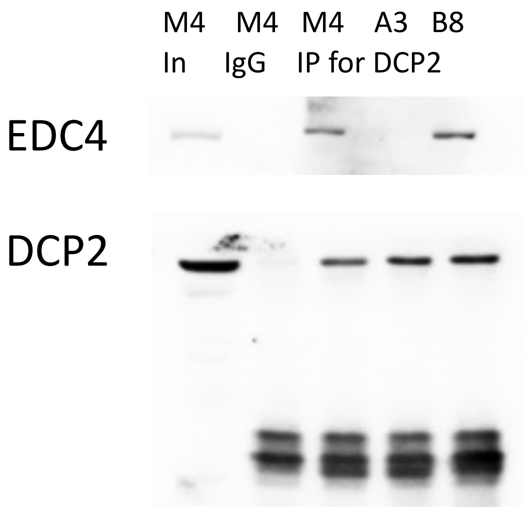

#replica 2 for Supplemental Figure 5
Superose 6 fractionation performed as described in the paper.
HCT116 cell line - this was one of our crispr control cell lines, different cell line that what was used in Supplemental Figure 5, DCP1a and DCP1b levels are not manipulated in these cells.
IN - is input sample used as control, HCT116 cells lysates

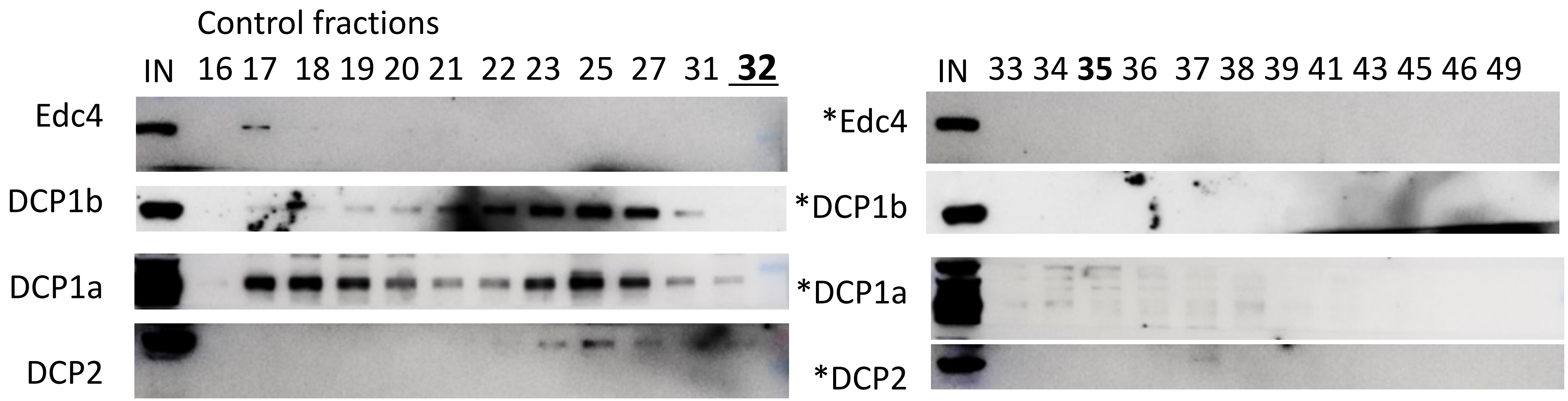

Approximate molecular weight by fraction:

32	--- 670 kDa
35	--- 158 kDa
38-39	--- 44 kDa
43-44	--- 17 kDa
51?	------------1.35 kDa

#extra data provided for DCP1a and DCP1b KO cells (AB) for double knock down, see response to reviewers' note
HCT116 cells were transfected with Cas9 protein and no gRNA to create control cells or Cas9 protein and a mix of sgRNAs targeting either DCP1a or DCP1b. Three days post transfection cells were transferred to a new plate and cultured for ten days. WB showing inputs. PARP and GAPDH levels were assessed.

Pool cells KO cells: DCP1a(A), DCP1b(B) and DCP1a and DCP1b(AB)
Untreated (Ut), 24 hrs, and 48 hrs 5 fluorouracil (5FU) treatment

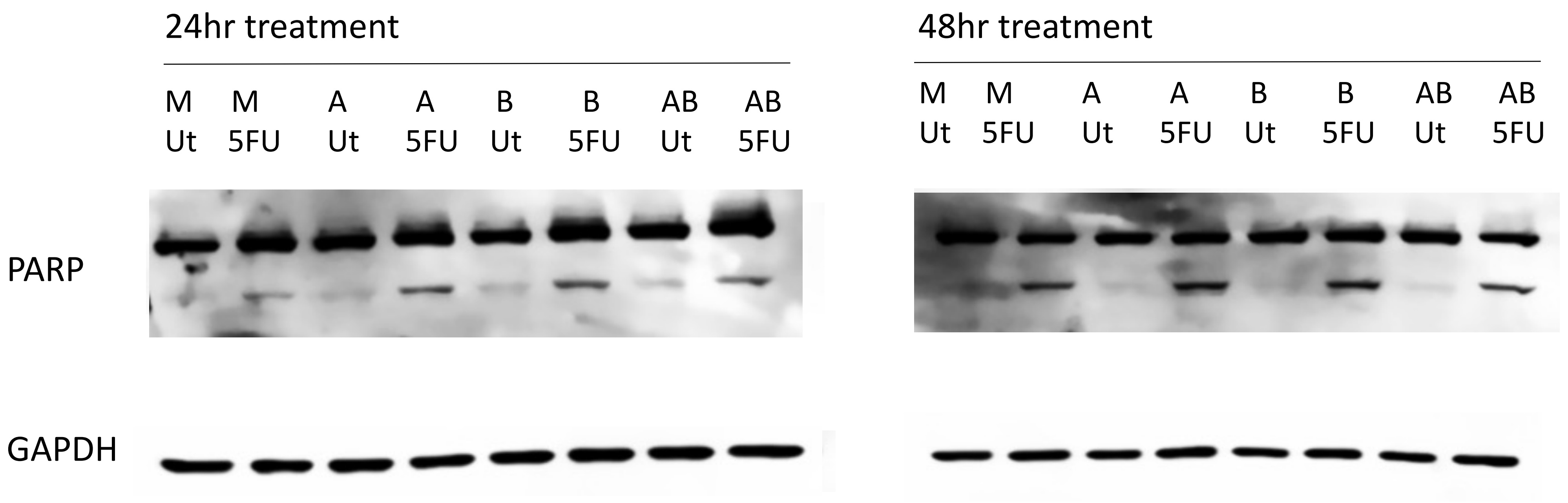

Supplement: Supplementary file 3 [file LSA-2024-02938_SdataF1.2_FS5.pdf]
